# Health Monitoring of Offshore Wind Structures: Sensing Technology, Uncertainty, and Artificial Intelligence

**DOI:** 10.3390/s26154697

**Published:** 2026-07-23

**Authors:** Ruixin Li, Qiang Liu, Xu Han, Xin Li

**Affiliations:** 1 State Key Laboratory of Coastal and Offshore Engineering, Dalian University of Technology, Dalian 116024, China; liruixin@mail.dlut.edu.cn; 2 Institute of Earthquake Engineering, School of Infrastructure Engineering, Dalian University of Technology, Dalian 116024, China; 3 Ningbo Key Laboratory of Integrated Development and Safety Assurance for Deep-Sea Energy and Resources, Ningbo Institute of Dalian University of Technology, Ningbo 315016, China; 4 PowerChina Huadong Engineering Corporation Limited, Hangzhou 311122, China; liu_q@hdec.com; 5 Zhejiang Huadong Mapping and Engineering Safety Technology Co., Ltd., Hangzhou 310014, China

**Keywords:** offshore wind turbines (OWTs), sensors, health monitoring, uncertainty, artificial intelligence, digital twin

## Abstract

Offshore wind farms are rapidly expanding into deeper and more remote ocean regions. Their structural safety and operational reliability in harsh marine environments have garnered widespread global attention. Sensing technologies capture structural and environmental conditions and are indispensable to structural monitoring. Accordingly, this review examines the applications of environmental monitoring, supervisory control and data acquisition, condition monitoring, and structural health monitoring systems covering both the horizontal-axis and vertical-axis types of fixed and floating offshore wind turbines. It also summarizes key technologies for data transmission and optimal sensor placement. However, uncertainty in sensing data can significantly affect monitoring results, yet existing studies lack an adequate summary and in-depth discussion. We therefore focus on sources of sensing uncertainty, including the marine environment, the host platform, variations in environmental and operational conditions, and sparse sensing. By analyzing their effects on monitoring data, we explore key methods for overcoming data uncertainties and improving sensing accuracy. This paper also evaluates the application potential of cutting-edge artificial intelligence and digital twin technologies. Furthermore, the study points out that fusing multi-source signal data to establish a highly reliable intelligent decision-making and early warning framework is likely to become an important development direction for offshore wind power monitoring. This review aims to provide valuable support for the safe development of offshore wind farms towards deep-sea regions over the coming decades.

## 1. Introduction

In recent years, driven by fossil fuel depletion and escalating carbon emission concerns, the global energy landscape has rapidly transitioned toward clean and low-carbon renewable sources. Wind energy has experienced exponential growth due to its abundant resources and maturity of relevant technologies. According to industry forecasts, the global cumulative installed wind power capacity was projected to reach approximately 1299 GW by the end of 2025 [1]. Due to the saturation of onshore wind resources and increasingly stringent land constraints, wind energy development is progressively shifting towards offshore regions [2,3]. During the same period, the global cumulative offshore wind capacity exceeded 92.3 GW, signaling a new phase of rapid, large-scale, and deep-sea development.

Beyond wind loads, offshore wind turbines (OWTs) must withstand complex marine environmental conditions, including waves, ocean currents, and seabed scour, rendering them more vulnerable to structural failure and damage. Generators and gearboxes frequently suffer from lubrication issues and bearing damage, driving the high failure rates of turbine drivetrains. For slender blades, transverse operational loads induce severe vibrations and strains. This directly accelerates fatigue accumulation and crack propagation. Furthermore, cyclic wind and wave loads trigger fatigue across critical components like the tower, foundation, mooring systems, and connecting bolts. Such localized fatigue can ultimately escalate into structural failures. This may cause overall unit instability or even turbine collapse, leading to prolonged unplanned downtime and power interruptions.

Due to the high cost of wind turbine structures, any component failure incurs substantial economic losses associated with prolonged downtime, maintenance, and replacement [4,5]. Therefore, to guarantee optimal power generation efficiency and overall system safety, lifecycle operation and maintenance has garnered significant industry attention. Statistics indicate that Operations and Maintenance (O&M) costs constitute a substantial proportion of total project expenditures, typically accounting for 25% to 30% [6]. To reduce these expenditures and optimize maintenance strategies, establishing highly reliable and technologically mature monitoring systems is crucial. Currently, comprehensive monitoring frameworks for large marine structures have been initially established. These frameworks primarily integrate environmental monitoring, supervisory control and data acquisition (SCADA), condition monitoring (CM), and structural health monitoring (SHM). For example, in 2016, the Block Island Wind Farm in the United States deployed an SHM system on operational wind turbines. This system incorporated wired and wireless accelerometers, strain gauges, inclinometers, and dedicated data acquisition units [7]. Mittelmeier et al. conducted wake analyses utilizing SCADA data from three offshore wind farms. They effectively combined these datasets with environmental measurements obtained from Light Detection and Ranging (LiDAR) and meteorological masts [8].

Despite significant advancements in modern monitoring technologies, the uncertainty and long-term stability of sensing systems remain pressing challenges in complex and dynamic real-world applications. During data acquisition and processing, conventional sensing technologies inevitably encounter various disturbances [9]. These sources of uncertainty primarily stem from marine environmental impacts, internal hardware degradation, hosting platform dynamics, varying operational conditions, and the sparse spatio-temporal sampling of sensors [7,10,11]. To address these challenges, recent research trends have shifted toward data-driven intelligent processing methods, particularly the deep integration of artificial intelligence (AI) [12]. By employing advanced AI algorithms like deep learning (DL) and reinforcement learning, researchers can extract robust feature representations from massive noisy datasets [13]. This approach significantly enhances the noise resistance and adaptability of systems under harsh conditions. Furthermore, it offers a novel paradigm for mitigating multi-source uncertainties.

Currently, there are already some literature reviews on wind turbine monitoring technologies. The research scope has comprehensively covered blade damage detection technologies and established a four-stage statistical pattern recognition paradigm ranging from operational evaluation to model development [14,15]. Lian et al. [16] examined OWT SHM systems, focusing on data acquisition, feature extraction, and identification. Additionally, they briefly discussed wind turbine reliability analysis and safety assessment methods. Yang et al. [17] reviewed SHM and fault diagnosis of OWT support structures, focusing on towers and foundations. Tong et al. [18] thoroughly analyzed the challenges faced by horizontal-axis OWTs in harsh marine environments. They concluded that monitoring systems integrating AI and machine learning (ML) represent the future direction of development. However, existing review papers rarely address the reliability issues associated with sensing data. Furthermore, there is a lack of a comprehensive synthesis regarding the application of AI and digital twin (DT) technologies within the offshore wind sector. In view of this, this paper provides a detailed review of sensing technologies for fixed and floating offshore wind turbines (FOWTs), including both horizontal-axis and vertical-axis configurations, focusing specifically on the sources of sensing uncertainty and the associated digitalization processes. Throughout this review, two fundamental questions are explored. First, how can the accuracy and fidelity of sensing data be improved in complex marine environments? Second, how can effective cost control be achieved for OWT monitoring systems? These questions are addressed by summarizing several critical research areas within the wind energy domain.

The contributions of this review are threefold. First, it synthesizes advances in sensing technologies, data transmission, multi-source data fusion, and sensor placement, clarifying their roles in OWT monitoring. Second, it summarizes the principal sources of sensing-data uncertainty and provides guidance on selecting suitable uncertainty quantification and data correction methods. Third, it compares representative AI approaches and examines how DT supports sensor data integration, condition assessment, and maintenance decision-making.

The remainder of this paper is structured as follows: Section 2 presents the review methodology. Section 3 details the classifications and applications of sensors utilized in OWTs. Section 4 describes sensing uncertainty and discusses its correction and quantification methods. Section 5 reviews the integration of AI within the structural monitoring domain. Section 6 explores the current application status of multidimensional DT technologies. Finally, conclusions are drawn in Section 7.

## 2. Methodology

Given the interdisciplinary nature of sensing and monitoring for OWTs, this review adopted a progressive, multi-stage search strategy. First, combinations of keywords related to sensor, OWT, monitoring, and uncertainty were searched in Web of Science, Scopus, and Google Scholar. The search covered titles, abstracts, and keywords from January 2010 to June 2026. English-language journal articles, conference papers, books, and technical reports were considered. Studies were retained if they focused on sensing applications in offshore wind, investigated OWT structures rather than individual electrical components, and reported a clear methodological framework, experimental design, case validation, or results. Duplicate and marginally relevant records were excluded.

To balance depth and coverage, the search was subsequently extended to accelerometer-, strain-, and fiber-optic-based sensing, as well as ML, life prediction, and safety assessment. Relevant studies on onshore wind turbines, ships, and offshore photovoltaic structures were also considered where their methods were applicable to offshore wind monitoring. In total, 214 publications were included in this review.

## 3. Sensing of Offshore Wind Turbines

### 3.1. Classification and Application of Sensing Technologies

This section details the classifications and applications of various sensors deployed in OWTs. Comprehensive monitoring requires the deployment of diverse sensors to track ambient environmental conditions, internal mechanical states, and structural health. Table 1 provides an overview of the standard sensors utilized in offshore wind monitoring. Specifically, it delineates the sensing equipment, core advantages, inherent limitations, and typical deployment locations corresponding to different monitoring targets. These targets span the domains of environmental monitoring, condition monitoring (CM), SHM, and related fields. Ultimately, these comparisons elucidate the critical role of monitoring data in evaluating turbine safety.

#### 3.1.1. Environmental Monitoring

Accurate marine environmental data are fundamental to ensure the safe operation of OWTs. Real-time observation of key environmental factors, such as wind, waves, and currents, enables the effective evaluation of environmental loads exerted on the turbines. This evaluation provides reliable data and a solid decision-making basis for the safe scheduling and stable power generation of wind farms.

Wind field monitoring primarily focuses on two parameters, i.e., the wind speed and direction at the locations of interest. Through traditional sensors such as offshore meteorological masts, nacelle anemometers, and buoys, the single-point wind conditions can be measured. Ultrasonic and hot-wire anemometers are usually used at extreme wind speeds. LiDAR systems can measure wind profiles, including wind speeds and directions at multiple heights. Between 2014 and 2016, LiDAR was mounted on a buoy to measure offshore wind speeds near Monhegan Island. Its built-in correction software compensated for the structural motion of the buoy itself, and the measurement results were consistent with land-based LiDAR [52].

Wave buoys can be applied to estimate the significant wave height, spectral peak period, and directional wave spectrum. They are commonly used monitoring tools in marine engineering, but are limited to single-point wave observations. Wave gauges and air-gap sensors also could serve the same function. Acoustic Doppler Current Profilers (ADCPs) provide flow velocity information based on the Doppler shift principle, and are widely used to measure wave spectrum parameters [53,54]. ADCPs have multiple variants, and scholars continuously improve the configuration of slanted and vertical beams to obtain the best measurement scheme [55]. Among these variants, the vertical beam is used for high-resolution surface wave tracking, while the slanted beam measures the directional wave spectrum. The radar sensing systems, including X-band radars [28] and high-frequency radars [56], can measure the surface waves by utilizing the propagation paths of radio waves. Sometimes, they can also extract information on wind speed, direction, and flow velocity. In addition, based on satellite remote sensing, marine data can be captured globally. However, constrained by the operating orbit, it still suffers from inaccurate estimation of the wave period and direction, with a limited sampling frequency.

#### 3.1.2. Supervisory Control and Data Acquisition

As a monitoring system for OWTs, SCADA is integrated into the nacelle controller to monitor and record long-term environmental parameters and turbine operational data, including wind speed, direction, gearbox and generator temperatures, rotor speed, yaw, pitch angle, and output power. SCADA operates at a relatively low sampling rate, and the analyzable data used in engineering practice typically consist of 10 min statistical values (maximum, minimum, mean, and standard deviation) for various parameters. Almost all commercial OWTs are equipped with a SCADA system as standard. The alarm system could be triggered when the critical data from SCADA exceed predefined thresholds. However, under complex dynamic loading conditions, fixed threshold settings are prone to false alarms, potentially increasing the O&M costs of wind farms. In addition, the reliability of the environmental data recorded by SCADA over the turbine’s service life is viewed with skepticism. For example, the nacelle anemometers are subject to interference from the rotor axial induction effect and flow around the nacelle [14]. Furthermore, the aerodynamic wake and local flow field distortion generated by rotor rotation cause deviations in the wind direction measured by the nacelle wind vane [57]. Therefore, the raw SCADA environmental signals should be corrected and calibrated for systematic errors before being used as calculation inputs.

Evaluating the health status of OWTs using SCADA data is considered a highly cost-effective and promising approach. Virtual sensing technology based on SCADA data, accelerometers, and strain gauges [58] has been applied to OWTs. Vieira et al. [59] proposed a regression model framework based on SCADA monitoring data, which performs well in predicting the remaining useful life (RUL) of the main bearings in wind turbines. Kong et al. [60] proposed a SCADA-based data-driven model. It was demonstrated that predicting the failure rates of wind turbines using SCADA operational databases is more objective and accurate than relying on the subjective judgments of experts. It is worth noting that although SCADA-based CM is advancing [61], its sampling rate remains too low for comprehensive CM and fault diagnosis. Furthermore, it is relatively difficult to obtain SCADA datasets with sufficient sample sizes. Therefore, professional and accurate dedicated condition monitoring systems (CMSs) are still required at present.

#### 3.1.3. Condition Monitoring Systems

The power generation systems of OWTs include components such as the rotor, gearbox, generator, and drivetrain. These mechanical and electrical components are prone to failure during operation. CMSs can assess the health condition of the components and perform fault detection through continuous real-time monitoring. In addition to the SCADA data introduced in Section 3.1.2, CM often relies heavily on external sensors to directly capture high-resolution response signals of the components. Vibration-based analysis commonly uses accelerometers, displacement sensors, and velocity sensors to monitor bearings and gearboxes [35]. Oil debris monitoring can effectively reflect the true wear status of gearbox components through the analysis of abrasive particles in the lubrication system [62]. Common non-contact sensors used in CM include infrared thermal imagers for temperature measurement and acoustic emission (AE) sensors for capturing high-frequency elastic waves.

CMSs have been widely applied in the safety assessment of OWT mechanical equipment. Relying on relevant physical sensors enables early fault warning and predictive maintenance [63]. However, the cost of sensor deployment restricts the development of CM technology. Simultaneously, with the rapid growth in the volume of offshore wind monitoring data, the traditional data analysis relying on manual diagnostic decision-making is incapable of meeting practical demands. These challenges are driving CM technologies to evolve toward low-cost and intelligent solutions.

#### 3.1.4. Structural Health Monitoring Systems

SHM systems aim to obtain structural response characteristics by analyzing monitoring data, thereby evaluating the damage status of critical structural components. Initially, SHM mainly served large hydraulic structures, high-rise buildings, and bridges, and later expanded and evolved into marine engineering structures. Due to the more complex service environments in deep-sea and far-offshore areas, the monitoring requirements for OWTs are more stringent. SHM in the offshore wind sector covers blades, towers, support structures, bolted joints, and mooring systems. To maintain a consistent technique-oriented classification, the following subsections are organized according to vibration, strain and fiber bragg grating (FBG) sensing, acoustic emission and ultrasonic inspection, machine vision, and load and inclination monitoring.

##### Vibration Monitoring

Structural damage in wind turbines leads to local stiffness attenuation, which in turn affects the local vibration characteristics, natural frequencies, and mode shapes of the structure. By deploying accelerometers on the blade surfaces, towers, and foundations, vibration acceleration signals can be obtained to analyze changes in modal characteristics. Based on their measurement principles, accelerometers can be classified into optical, capacitive, piezoelectric, and piezoresistive types. Common examples include fiber optic and Micro-Electro-Mechanical System (MEMS) capacitive accelerometers. The former features high sensitivity and immunity to electromagnetic interference. However, the high price of optical components restricts the large-scale application of fiber optic technology in the acceleration monitoring of deep-sea structures. MEMS accelerometers offer advantages such as low cost, lightweight, and simple installation. They are suitable for monitoring low-frequency vibrations and are widely used in tower and foundation vibration monitoring. However, the vibration measurements obtained by MEMS accelerometers are highly susceptible to environmental fluctuations. Their installation is therefore important for reliable monitoring. It is also worth mentioning that the measured accelerations largely contain the global dynamic effects. Consequently, it is possible to detect structural status changes away from the accelerometers. However, this characteristic limits their ability to identify minor local damage at an early stage.

Ge et al. [64] developed a dual-axis intensity-modulated optical fiber accelerometer. Experimental results demonstrated that this optical fiber accelerometer exhibits robust performance and can meet the monitoring requirements for low-frequency vibrations (typically below 10 Hz) in wind turbine blades. Sakaris et al. [65] utilized the response signal from a single accelerometer to investigate damage detection in floating wind turbine tendons under varying environmental conditions. Zhou et al. [66] utilized wireless triaxial accelerometers deployed on the tower and monopile to collect the vibration responses of an OWT under real sea conditions. A weak modal identification method was introduced to process these acceleration data, thereby extracting the weak structural vibration modal characteristics more effectively. Zhang et al. [67] developed an OWT blade monitoring method based on single-channel vibration signals, evaluating the blade condition by analyzing the linear regression between the first-order natural frequency and rotational speed. The study further pointed out that in the low-speed range of the wind turbine, only a limited amount of vibration data is required to achieve blade fault diagnosis.

Rao et al. [68] adopted a Bayesian spectral decomposition method to perform operational modal analysis of monopile OWTs based on vibration data obtained from a uniaxial accelerometer on the tower. Using acceleration records collected over four years from a full-scale vertical-axis wind turbine, Pagnini and Piccardo [69] identified modal frequencies and damping ratios under parked and operating conditions. Relying on the transverse vibration response obtained from the nacelle accelerometer, Skrimpas et al. [70] proposed a wind turbine blade icing detection method that combines vibration and power curve analysis. Wang et al. [71] combined finite element analysis and mode shape curvature difference information to compare the modal parameters and dynamic response characteristics of blades under excited vibration, ultimately identifying blade damage.

##### Strain Monitoring

Compared to displacement or acceleration monitoring, strain-based monitoring reflects local damage more intuitively and accurately. Commonly used strain sensors include strain gauges and optical fiber sensors. An electrical resistance strain gauge typically consists of a polymer insulating backing layer embedded with grid-like metal wires or foils, which is used to monitor strain and deformation at critical structural positions. In blade monitoring, strain gauges are often attached to the blade root and the maximum chord surface, or directly embedded into the fiber layers of the composite blades. For support structure monitoring, they are primarily deployed at typical stress concentration points, such as the joints between the tower and the underwater tubular joints of the jacket foundation. Because strain measurement relies on point-wise sensing techniques, its reliability depends heavily on the sensor placement. Specifically, the closer the sensors are to the damage area, the higher the measurement accuracy. Compared to traditional electrical resistance strain gauges, optical fiber sensors feature immunity to electromagnetic interference and high sensitivity, offering significant advantages for long-term continuous monitoring. Among optical fiber sensors, Fiber Bragg Grating (FBG) sensors are the most extensively researched and widely applied. FBG sensors capture variations in temperature and axial strain through shifts in the Bragg wavelength over time. The calculation of the Bragg wavelength is expressed as Equation (1) [72]:

(1) $$\lambda_{B}=2\cdot n_{\mathrm{eff}}\Lambda$$

where $\lambda_{B}$ is the Bragg wavelength; $n_{\mathrm{eff}}$ is the effective refractive index of the fiber core; Λ is the grating period.

Pacheco et al. [73] explored a full-lifecycle fatigue assessment method for wind turbine blades based on an FBG strain sensing network. Wen et al. [74] developed an online monitoring system for strain and load on floating wind turbine blades based on FBG and fiber optic rotary joints. Test results demonstrated that this system can accurately capture the dynamic strain responses induced by incident waves and platform pitch resonance. Guo et al. [75] utilized measured strain data to analyze the magnitude and distribution characteristics of strain in monopile foundations. Ma et al. [76] employed FBG sensors to monitor the axial strain between the connecting sections of the underwater foundation, achieving highly accurate measurements of underwater cross-sectional loads. Zhu et al. [77] proposed a strain sensing module that pre-packages FBG sensors within carbon fiber-reinforced polymers for monitoring the strain of blades. Van Esbeen et al. [72] used cascaded FBG sensors to monitor the bending deformation of a vertical-axis wind turbine tower.

Accurate condition assessment of wind turbine structures requires obtaining the true stress distribution along the entire structural length and at critical fatigue hotspots. However, installing contact strain sensors at certain fatigue nodes, such as the underwater mudline of monopile foundations, is typically unfeasible. To address this limitation, the method of full-field strain reconstruction for OWTs has been investigated. This process utilizes sparse strain gauge data from accessible locations above and below the waterline, integrated with accelerometers and calibrated finite element models. Iliopoulos et al. [78] proposed a multi-band modal decomposition and expansion technique. This method uses limited strain and acceleration data from the upper and middle tower sections. It divides the structural response in the frequency domain into three independent regions, i.e., the quasi-static, low-frequency dynamic, and high-frequency dynamic regions. Henkel et al. [79] extended this method to jacket foundations. It was pointed out that due to the prominent local modes of the jacket braces, relying solely on the limited measurements from the tower will introduce significant errors in the fatigue prediction of X-joints. This indicates that the reconstruction of complex multi-node foundations still requires further technological breakthroughs.

Strain-based techniques are also applicable to the condition assessment of bolted joints. Li et al. [80] formulated a point-specific damage index using bending strain responses and proposed a regional method for detecting loose bolts in wind turbine flanges. Chen et al. [49] used FBG sensors to capture strain-gradient characteristics near crack tips, enabling the localization and inclination identification of micro-cracks in high-strength bolts.

##### Acoustic Emission Crack Monitoring and Inspection

**Figure 1:**
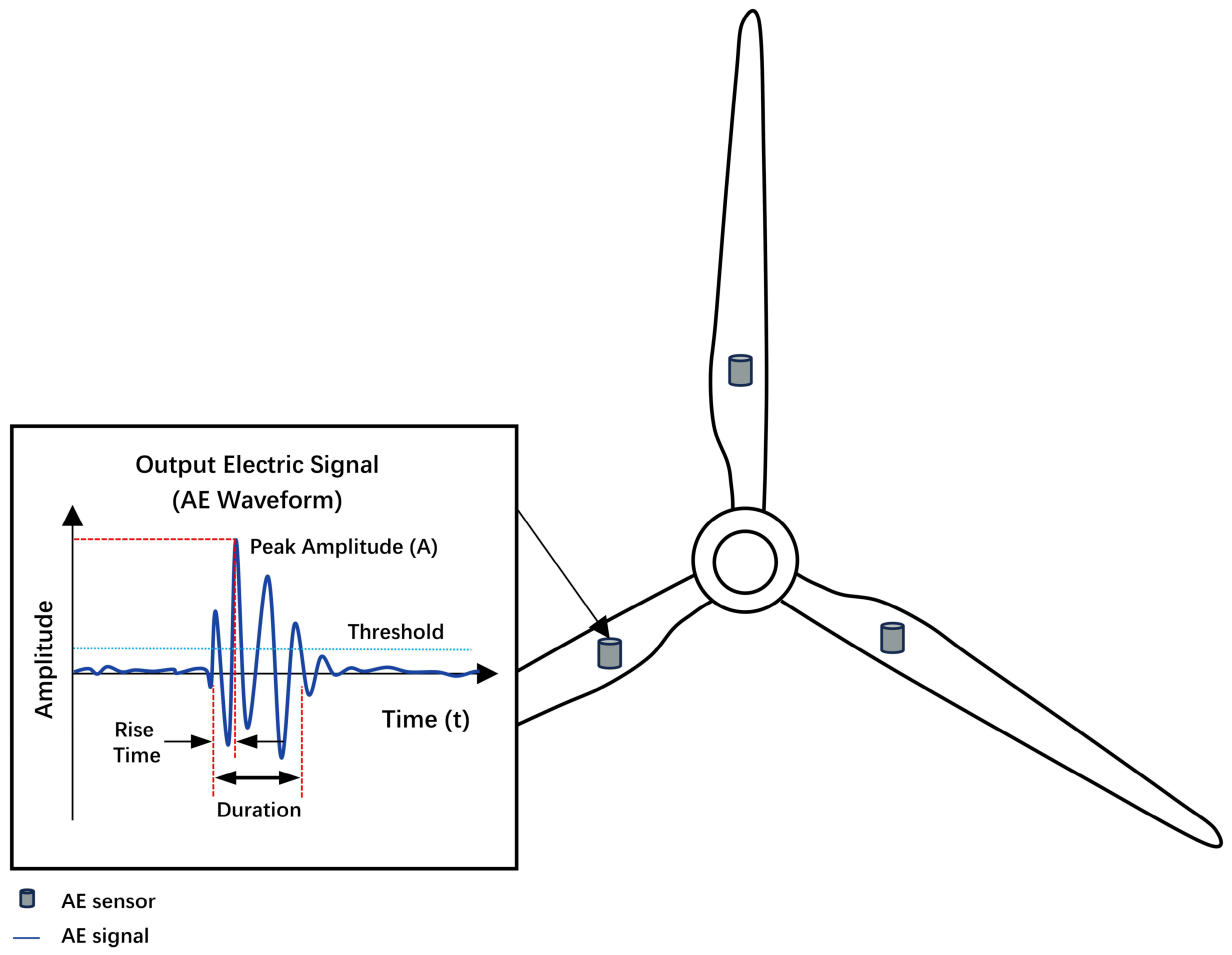
Schematic diagram of acoustic emission (AE) signal feature extraction.

In wind turbine blade detection, AE sensing technology has reached a relatively mature stage of application. High-frequency elastic stress waves are induced inside a structure with cracks, and the AE sensor detects blade damage by capturing these waves and converting them into electrical signals. An illustrative waveform of an AE signal and the definitions of its fundamental characteristic parameters (e.g., peak amplitude, rise time, and duration) are schematically shown in Figure 1. AE sensors cover a wide frequency range, have high measurement accuracy, and can identify micro-cracks appearing during the early stages of structural damage. The usually short duration of crack propagation can easily lead to sudden fracture failure of wind turbine blades; therefore, AE sensors play an irreplaceable role in inspections and monitoring of small crack initiation. Hüsken et al. [81] used AE technology to monitor the internal crack formation process within the grouted connection section of OWTs. Cheng et al. [82] proposed an AE monitoring method combining micro-piezoelectric sensors and a damage baseline. Experiments demonstrated that this method effectively overcomes the limitation of low signal-to-noise ratios in micro-sensors, accurately distinguishing between normal and damaged states, and significantly advancing the early warning time for damage compared to traditional methods. Jiang et al. [83] extended the application of AE technology from traditional damage monitoring to structural surface environmental anomaly detection; specifically, by deploying AE sensors on the inner wall of the blade, they constructed a real-time wind turbine blade icing monitoring system combining AE and deep learning.

During normal operation of wind turbines, the power generation system produces mechanical noise, while the rotating blades generate aerodynamic noise. These two types of noise affect the identification of high-frequency AE signals and interfere with the precise extraction of damage features. Therefore, it is necessary to eliminate the impact of environmental noise during the pre-processing of AE data. Bo et al. [84] developed a feature extraction method based on blind deconvolution, which successfully separated the characteristic frequencies of the micro-crack initiation and propagation stages from AE monitoring signals. Chen et al. [45] adopted the Db4 wavelet thresholding method to pre-process and denoise raw signals. By combining this with hybrid deep learning, they achieved the localization of AE sources within the wind turbine blade structure. As with other sensing technologies, the placement of AE sensors needs to be close to the damage point to ensure measurement accuracy. In addition, AE is fundamentally a passive sensing technology. For cracks inside the structure that are not in the active evolution stage, that is, cracks that have completed propagation or are temporarily not in the active propagation period, identifying damage requires a reference.

Unlike passive AE monitoring, active ultrasonic inspection identifies internal defects by analyzing the propagation and reflection of externally generated waves. For bolted joints, Sun et al. [85] developed an ultrasonic phased-array system based on a Fermat spiral configuration to detect micro-cracks at bolt roots.

##### Machine Vision

Driven by continuous advancements in computer image processing, machine vision technology has developed rapidly and has been widely applied in engineering monitoring. Vision-based measurement is a non-contact sensing technology that eliminates the need for sensor installation on the turbine’s structural surface, enabling remote and full-field deformation monitoring. Visual monitoring of OWTs primarily utilizes high-definition cameras, infrared thermographic cameras, or binocular stereo vision systems—frequently deployed via Unmanned Aerial Vehicle (UAV) platforms—to acquire sequential images of target components. By integrating advanced digital image processing techniques, such as digital image correlation or target tracking, alongside ML algorithms, image data from various angles are utilized to detect surface defects and calculate the actual dynamic displacement of the structure. Compared to traditional point-based sensors, such as Global Navigation Satellite System (GNSS) receivers or inclinometers deployed at the tower top, these vision-based methods exhibit high spatial maneuverability and avoid complex physical installations in harsh marine environments.

Yang et al. [86] proposed a machine-vision-based method considering the dynamic updating of displacement conversion coefficients, achieving high-precision monitoring of the dynamic displacements during biaxial fatigue tests of wind turbine blades. Dutta et al. [87] developed a dual-camera visual monitoring system mounted on an unmanned inspection vessel for autonomous tracking and high-resolution image acquisition of blades during OWT operation. This system utilizes a convolutional neural network (CNN) object detection model and image segmentation technology to extract blade features in real time and predict their dynamic trajectories. Compared to traditional fixed measurement systems, UAVs have emerged as novel platforms that stand out in large-scale structural visual monitoring due to their spatial maneuverability and comprehensive field-of-view coverage. Li et al. [88] developed a UAV-based monitoring method using a target-free vision algorithm incorporating the discriminative scale space tracker to extract the dynamic characteristics of wind turbine blades, while eliminating UAV hovering drift errors through high-pass filtering and an adaptive scale factor. Wang et al. [89] proposed a data-driven visual inspection method that utilizes extended Haar-like features and a multi-model cascade classifier to achieve precise localization and recognition of blade cracks. Fontenla-Carrera et al. [90] developed a collaborative system involving support vessels and multiple UAVs to perform inspection tasks for offshore wind farms. Moreno et al. [91] constructed a CNN-based visual damage detection system that utilizes robots to collect images along the blade surface, completing the automatic recognition of specific defects such as lightning strikes, wear, and fractures.

Machine vision can also identify bolt loosening by tracking changes in nut rotation. Park et al. [92] extracted nut rotation angles from images to determine bolt-loosening states.

Machine vision breaks through the limitations of physical sensor placement, providing intuitive monitoring results that are unaffected by structural temperature changes. However, at the current stage, visual algorithms are mostly limited to single specific tasks, resulting in a lack of generalization capability. Furthermore, continuous high-frequency image acquisition generates massive volumes of multidimensional data, which significantly complicates subsequent data processing.

##### Load and Inclination Monitoring

**Figure 2:**
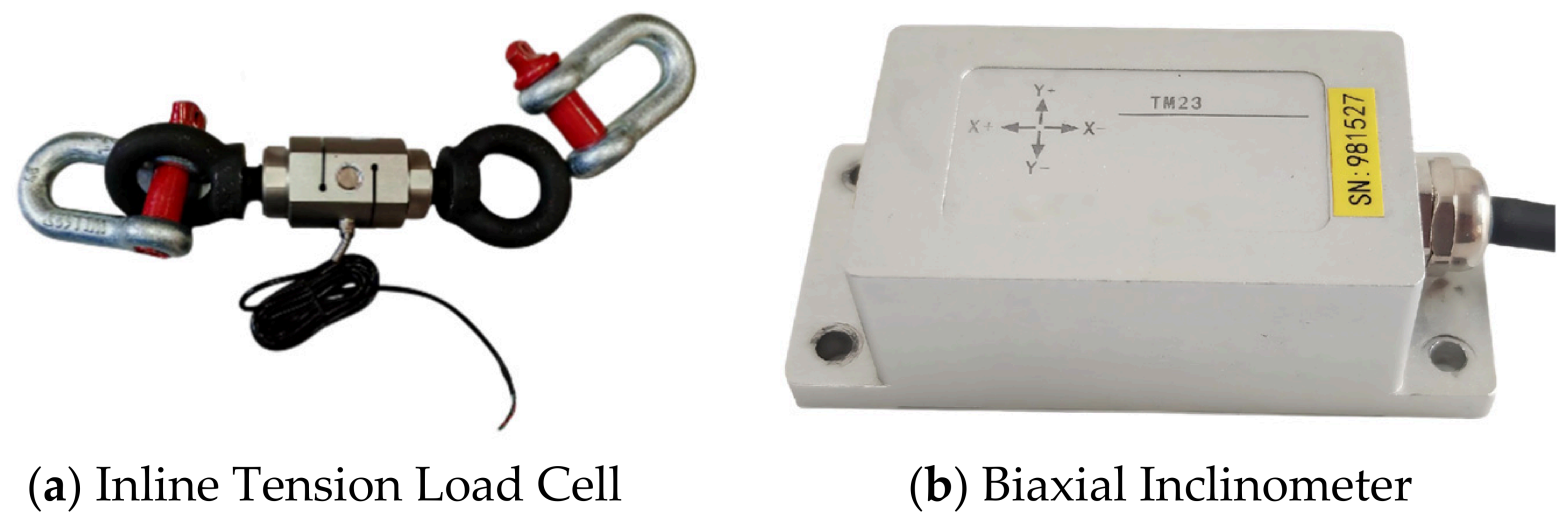
Sensors for floating offshore wind turbine (FOWT) mooring system monitoring.

Load cells and inclinometers provide direct or indirect measurements of tensile forces and angular responses. In offshore wind monitoring, these techniques are particularly relevant to the tendons and mooring lines of FOWTs, which maintain platform position and limit horizontal offsets. A typical mooring monitoring scheme obtains continuous, long-term mooring responses by installing tension sensors (Figure 2a) and inclinometers (Figure 2b) at critical locations. Although this method is intuitive and efficient, it fails to measure the variations in tension along the whole mooring length, nor can it accurately capture suddenly increased mooring tensions. Chung et al. [51] utilized local inclination and top tension data from tension leg wind turbine tendons; based on finite element interpolation and the small inclination assumption, they inversely estimated the tension and bending moment distribution over the full length of the tendon from discrete measurement points.

Considering the high cost and difficulties associated with the installation and maintenance of underwater sensors, some researchers predict the health state of the mooring system using responses from low-cost and easily deployed sensors. Yang et al. [93] adopted a time delay neural network to establish a nonlinear mapping between the six-degree-of-freedom motion of the floating body and the mooring tension. Payenda et al. [94] utilized platform motion data in conjunction with a recurrent neural network (RNN) model to predict the dynamic tension of the mooring system in a semi-submersible wind turbine. The bidirectional long short-term memory (BiLSTM) model was reported to achieve the highest prediction accuracy by comparing the performance of different deep learning algorithms under various environmental load conditions. Coraddu et al. [95] constructed a segmented health state prediction framework for mooring lines based on tower accelerometer data and weakly supervised learning. The results showed that under the most challenging weakly supervised scenario, the prediction error of this data-driven model was about 4%, effectively confirming the feasibility of using common sensors to replace traditional tension sensors for tension monitoring.

Overall, different monitoring tasks require different types of sensing information. Accelerometer-based monitoring is suitable for the continuous monitoring of global vibration responses, whereas strain monitoring is primarily used for local strain measurement and fatigue assessment. AE monitoring focuses on detecting active damage processes, while UAV-based visual inspection is suited to periodic surface inspection. For floating platforms, GNSS is mainly used to monitor platform position and motion, whereas load cells and inclinometers support mooring-line tension monitoring.

In addition to monitoring objectives, the technology readiness level (TRL) is an important criterion for selecting offshore monitoring solutions. Relevant technical reports assess standard SHM systems incorporating accelerometers, electrical resistance strain gauges, and inclinometers at TRL 9 [96], indicating validation in an operational environment. UAV-based visual inspection and GNSS-based position monitoring also exhibit relatively high technology readiness. By contrast, AE monitoring has a lower TRL, and its applicability under offshore operating conditions requires further validation.

### 3.2. Data Acquisition and Transmission Methods

Monitoring of OWTs at high sampling rates inevitably generates massive multi-source data, posing a significant challenge for the limited bandwidth and high latency communication links. The data communication network is mainly composed of a collaborative integration of wired transmission and wireless sensor networks [97]. Mainstream wireless technologies applied to OWT SHM encompass short-distance local transmission technologies (Wi-Fi and Zigbee) and low-power wide-area network technologies, including Long Range (LoRa) and Narrowband Internet of Things (NB-IoT), which exhibit significant differences in transmission distance, bandwidth, and power consumption. Table 2 compares these technologies in terms of data rate and bandwidth, transmission distance, power consumption, and reliability, providing a basis for selecting appropriate communication links for local batch transfer and long-distance offshore data transmission. Given the advantages and disadvantages of different communication protocols, current engineering practices tend to build multi-tier wireless transmission networks. The bottom layer uses Wi-Fi or Zigbee to establish a high-speed local data acquisition network inside the wind turbine, while the upper layer utilizes LoRa, NB-IoT, fourth-generation Long-Term Evolution (4G LTE), or fifth-generation (5G) networks—combined with submarine optical cables—to achieve long-distance, high-reliability trunk transmission from the wind farm to the onshore centralized control center.

Based on variations in data types and communication conditions, data transmission usually adopts a tiered processing and transmission mechanism. Low-frequency SCADA operation data and key transient fault alarm information can be directly transmitted to the shore in real time through the communication link. Conversely, due to the massive volume of high-frequency raw monitoring data, these datasets are usually stored locally and offline within the nacelle. Subsequently, when maintenance personnel or drones approach, high-speed batch extraction is achieved through a broadband ad hoc network.

In addition, with the development of edge computing technology, deploying embedded processing units at the sensing front end has become an important trend [98]. These units can complete data cleaning, noise reduction, and feature extraction locally, transmitting only low-dimensional damage features or status indicators to the shore base. This approach significantly reduces the communication load at the source and improves the system’s real-time performance and reliability.

Local data cleaning and anomaly screening determine whether anomalous measurements are removed before analysis, whereas denoising and feature extraction govern the structural information retained in processed signals [98,99]. Consequently, the quality of local processing directly affects the reliability of SHM, AI-based monitoring, and DT updating. Inadequate filtering, compression, or feature-extraction settings can suppress damage-sensitive information or introduce processing-induced bias. Edge algorithms should therefore be evaluated under representative operating conditions, with processing parameters and data-quality metrics documented to ensure traceability [100].

**Table 2:** Representative comparison of communication technologies for OWT monitoring.

Technology	Data Rate and Bandwidth	Transmission Distance	Relative Power Consumption	Indicative Reliability	Ref.
Wi-Fi	2–600 Mbps; 20/40 MHz	Typically ≤ 100 m	High	Moderate; line-of-sight- and interference-dependent	[101]
Zigbee Pro	250 kbps; approximately 2 MHz occupied bandwidth	Typically 10–100 m per hop; extendable through multi-hop networking	Very low	Moderate; topology- and routing-dependent	[101]
LoRaWAN	0.3–50 kbps (mode-dependent); commonly 125 kHz	Several kilometers; more than 10 km under favorable line-of-sight conditions	Very low	Moderate–high; distance- and interference-dependent	[102]
NB-IoT	Downlink data rate: approximately 60 kbps; uplink data rate: approximately 20–50 kbps; bandwidth: 180 kHz	Kilometer-scale; approximately 30 km modeled in a near-shore environment	Low; coverage- and repetition-dependent	High where cellular coverage is available	[103]
4G LTE	Several to tens of Mbps; channel bandwidth up to 20 MHz	Kilometer-scale; up to approximately 100 km for dedicated maritime networks	Moderate–high	High; coverage- and backhaul-dependent	[104]
5G	Tens to hundreds of Mbps; deployment- and spectrum-dependent	Kilometer-scale; 11.3 km reported in an OWT trial	Moderate–high	High within private-network coverage; backhaul-dependent	[105]
Subsea optical fiber based on an EPON	1 Gbps in the reported EPON design	20 km in the reported EPON design	Low per transmitted bit; active terminals required	High with full protection; modeled network downtime of approximately 39 min/year	[106]

Note: LoRaWAN, long-range wide-area network; NB-IoT, narrowband Internet of Things; LTE, long-term evolution; 4G, fourth generation; 5G, fifth generation; EPON, Ethernet passive optical network.

### 3.3. Multi-Source Heterogeneous Data Fusion Technology

Limited by inherent physical measurement mechanisms and observation dimensions, single sensors often yield monitoring results with high uncertainty. For example, vibration-based monitoring often lacks sufficient sensitivity and exhibits significant uncertainty [107]. Although vision-based monitoring is less affected by the environment than other monitoring methods, it is also limited by water clarity when conducting underwater monitoring [108,109]. Similarly, radar-based sensing is hindered by marine biofouling on the seabed [110,111]. Furthermore, given the significant high-dimensional and nonlinear characteristics of OWT dynamic responses, traditional single-load analysis methods struggle to comprehensively and accurately reflect structural health under the coupled actions of wind, waves, and currents [112]. To solve these problems, researchers comprehensively utilize the signal data collected during the operation of OWTs. Through combining vibration, strain, optical fiber, acoustics, SCADA and environmental loads, they adopt advanced data-driven algorithms to achieve the fusion of data signals.

**Figure 3:**
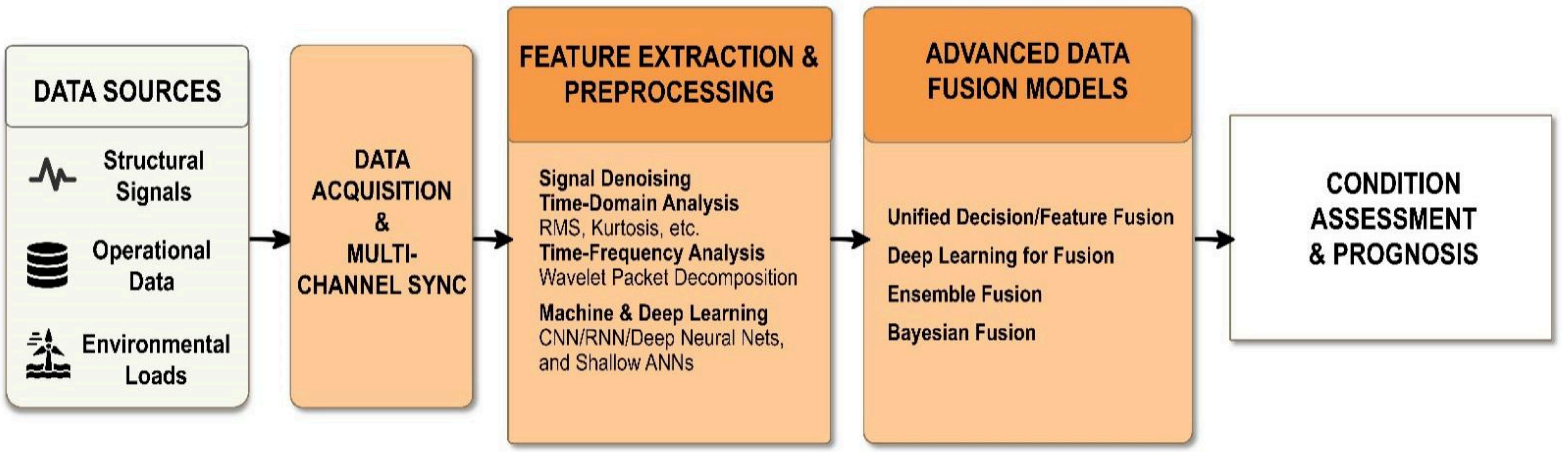
A generalized multi-source data fusion framework for condition assessment and prognosis.

To efficiently manage the multi-source data acquired from complex marine environments, a generalized framework for data fusion and condition assessment is conceptualized in Figure 3. For example, Petrovska et al. [113] utilized strain sensors installed in the transition piece of monopile OWTs, combining these measurements with SCADA and wave data to conduct a fatigue life assessment of the support structure. Relying on SCADA operational data and vibration acceleration signals, Wu and Su [114] employed nonlinear state estimation techniques to model key wind turbine components, achieving real-time condition prediction and fault identification. Sethi et al. [115] proposed a decision fusion framework taking vibration and acoustic signals as inputs; experimental results demonstrated that this heterogeneous data-based decision-level fusion method yields higher accuracy than single-source data approaches. Khan and Byun [116] developed a multimodal ML framework integrating regression and classification algorithms, showing that this multi-source fusion model can accurately predict and classify wind turbine faults 48 h in advance. Li et al. [117] fused high-frequency in situ strain data with low-frequency SCADA data, utilizing a multidimensional Archimedean Copula function to construct a joint probability distribution model for various heterogeneous loads. Xu et al. [118] constructed a DT-driven intelligent operation and maintenance framework that incorporates multidimensional SCADA parameters and employs an optimized attention-based temporal convolutional network for real-time monitoring and early warning of gearbox conditions.

Effective multi-source data fusion requires temporal harmonization of heterogeneous data. Sensor channels should be referenced to a common time base. Timing offsets induced by clock drift and communication latency can be estimated and corrected using cross-correlation or phase-matching techniques [119]. Signals sampled at different rates, such as high-frequency vibration signals and low-frequency SCADA data, can be aligned within common time windows through resampling, interpolation, or feature-level fusion. Missing data should be handled according to their distribution and duration [120]. Fusion weights should further reflect measurement variance, signal-to-noise ratio, data completeness, and sensor health status, thereby reducing the influence of unreliable observations [121].

OWT SHM is increasingly adopting multi-source heterogeneous data fusion [122]. By exploiting complementary sensor information, data fusion can improve the robustness and predictive accuracy of structural condition assessment. However, complex DL-based multimodal models may obscure the contribution of individual data sources and reduce model transparency [123]. Effective fusion for OWT SHM should therefore balance predictive performance with model transparency to support sound engineering interpretation and decision-making.

### 3.4. Sensor Layout Optimization

For contact-based monitoring, an insufficient number of sensors or suboptimal sensor placement may prevent complete acquisition of monitoring information [124,125,126,127]. However, simply pursuing a high-density sensor network will inevitably lead to a high data dimensionality and high cost [128,129,130]. Therefore, optimizing sensor array layouts to balance structural observability and cost has become an interesting topic to study for engineering applications [131]. Existing studies show that complementary sensing can improve load and model-parameter estimation [132], whereas optimization-based [133] and data-driven sensor selection [134] can reduce sensor redundancy while maintaining monitoring performance.

Intelligent search algorithms, including genetic algorithm (GA), particle swarm optimization, simulated annealing, and grey wolf optimization, are commonly used for discrete, nonlinear, and multi-objective sensor-placement problems. However, their results depend on the objective function and hyperparameter settings [135]. Information-theoretic methods quantify the residual uncertainty associated with different sensor layouts using information entropy [128,129].

For the operational modal testing of a 3 MW wind turbine, Schulze et al. [136] compared three accelerometer-placement methods: weighted off-diagonal element minimization, orthogonal–upper-triangular decomposition, and GA. Among the evaluated configurations, the GA-based sensor layout achieved the best performance.

These studies indicate that different sensor-placement tasks require different optimization criteria [134]. Modal observability is suitable for modal identification, information gain for parameter estimation, response-reconstruction error for virtual sensing, and fault-identification performance for condition monitoring and fault diagnosis [137]. Sensor-layout optimization should also consider the specific monitoring task, environmental variability, and deployment cost, rather than selecting sensor locations solely on the basis of modal response characteristics.

## 4. Sensor Data Uncertainty

**Figure 4:**
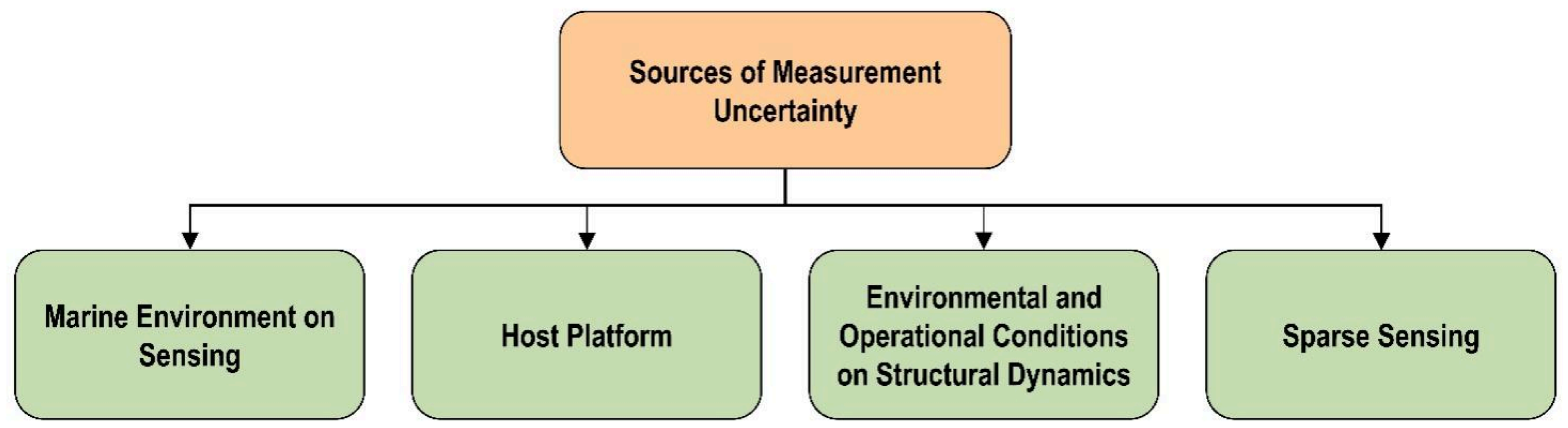
Sources of measurement uncertainty in marine engineering monitoring systems.

Affected by the marine environment and structural loads, the monitoring data exhibit significant inherent uncertainty and measurement errors. Ignoring these uncertainties can distort nacelle control strategies and bias fatigue load calculations, potentially causing an underestimation of the wind turbine’s ultimate limit state safety thresholds, which can be costly or even dangerous. Consequently, this compromises the reliability of O&M decision-making and life extension assessments for offshore wind farms. This section elaborates on the primary sources of measurement uncertainty in typical monitoring sensors, as shown in Figure 4, and briefly describes current methods for addressing them.

### 4.1. Impact of Marine Environment on Sensing

The complex marine environment inevitably introduces noise into monitoring data [138,139], while factors such as salt spray, humidity, and temperature fluctuations accelerate sensor corrosion and degradation. Research on the corrosion monitoring of OWTs indicates that temperature, salinity, and marine environmental fluctuations are key factors affecting monitoring accuracy and long-term reliability [140,141,142].

When monitoring data are used for load estimation, sensor-related errors may bias fatigue estimates. A laboratory study of a six-axis MEMS sensor reported a temperature-induced accelerometer bias of up to 0.023 g as the ambient temperature increased from 25 °C to 75 °C [143]. Long-term zero drift in strain-gauge measurements can reach approximately 100 με/year, equivalent to about 21 MPa/year [144]. Different treatments of strain-gauge zero drift and thermal stress resulted in up to an 11.3% difference in estimated cumulative fatigue damage for a wind turbine tower [145]. Additive zero drift primarily shifts the baseline of the strain signal. Its effect may therefore be limited when fatigue damage is evaluated solely from stress ranges without mean-stress correction. In contrast, sensitivity errors alter the signal amplitude and directly bias the stress ranges used for fatigue damage accumulation. Welded FBG sensors at the Northwind offshore wind farm required a sensitivity correction factor of 0.9 [146]. In a UAV photogrammetry experiment on a wind turbine blade, reducing illuminance from 200 lux to 65 lux increased the three-dimensional registration error and its standard deviation by approximately 60% and 63%, respectively [147].

The measured signals can potentially be corrected. This has led to a body of research on data preprocessing, including data cleaning and imputation to address noise and missing measurements [68,148]. Frequency-domain filtering, wavelet-threshold denoising, and signal-decomposition methods, including complete ensemble empirical mode decomposition with adaptive noise (CEEMDAN) and variational mode decomposition (VMD), can mitigate marine environmental noise. Statistical imputation is commonly used to reconstruct short-term missing data. Data-driven techniques also support the detection of sensor bias and drift. In particular, multivariate statistical methods such as principal component analysis (PCA) and partial least squares (PLS) have been applied for this purpose [149,150]. Temperature variations may additionally produce spurious components in sensor measurements; their influence can be reduced through temperature-coefficient correction or reference-temperature compensation. The representative metrics, advantages, and limitations of these uncertainty quantification methods are summarized in Table 3. Targeting fault diagnosis in noisy environments, Du et al. [151] proposed a method based on CEEMDAN and a hybrid neural network to effectively reduce noise and improve diagnostic accuracy.

### 4.2. Impact of Sensor Mounting Platforms

Non-contact sensors, such as GNSS, laser-based instruments, and microwave radars, are inherently sensitive to the dynamics of their mounting platforms. Borraccino et al. [159] pointed out that the wind speed accuracy of nacelle LiDAR depends on the installation attitude and observation geometry. This issue becomes even more pronounced on FOWTs. Gräfe et al. [160] rigorously demonstrated that floating foundation motion significantly affects the wind speed measurements of nacelle-mounted forward-looking LiDAR, necessitating the integration of motion measurement and compensation algorithms to recover accurate wind speed data. However, the performance of this analytical-model-based correction method depends on platform-motion parameters, LiDAR measurement geometry, and wind-field assumptions, and is influenced by the floating-platform pitch amplitude and wind-shear parameters. Similarly, the stability of vision-based monitoring is susceptible to environmental excitations, where wind-induced camera jitter directly degrades displacement recognition accuracy. Therefore, Liang et al. [161] adopted a 6-degree-of-freedom camera motion compensation method integrating dual inertial measurement units (IMUs) to decouple camera motion from tower motion. This method eliminates the need for fixed background reference points and additional cameras, making it more suitable for field applications in wind farms. Nevertheless, its compensation accuracy remains sensitive to IMU drift and site-specific imaging conditions.

### 4.3. Impact of Environmental and Operational Conditions (EOCs) on Structural Dynamics

OWTs operate in complex and varying marine environments over long periods [162]. Specifically, temperature fluctuations induce structural expansion and contraction, thereby altering internal stress, natural frequencies and mode shapes [163,164,165]. Wind-induced vibrations can adversely affect sensor measurement accuracy [166], and humidity variations can alter the material properties of blade composites and their adhesive interfaces [167,168]. Mass load effects such as the growth of marine organisms [169], icing [170,171], additional hydrodynamic mass [172], and floating wind turbine ballasting conditions will change the equivalent mass and boundary conditions of the overall structure, thereby affecting the measurements [7]. The impact of the environment on the structural dynamic characteristics is sometimes more obvious than the damage to the structure itself. Therefore, it is critical to identify such changes and improve the interpretability of the sensed data.

Analyzing long-term monitoring data from the DanTysk offshore wind farm under shutdown and idling conditions, Kjeld et al. [173] binned vibration samples according to wind speed, significant wave height, and wave period, subsequently employing a linear regression model to extrapolate damping and frequency parameters to a zero-wind-speed reference state. Avendaño-Valencia et al. proposed a Gaussian process (GP) model for the monitoring of in-service wind turbine blades to reveal the law of vibration characteristics changing with environmental and operational variables such as wind speed, rotational speed, and temperature in a healthy state. On this basis, samples deviating from the GP healthy prediction interval are regarded as potential damage responses [174]. Zhang et al. [175] utilized GP regression to model the correlations of monitoring features between adjacent healthy turbines, subsequently tracking the prediction residuals. Given that environmental and operational variations affect multiple units similarly, structural disturbances induced by environmental changes can be effectively mitigated by analyzing these relative features. Overall, methods that explicitly incorporate environmental and operational parameters can quantify the effects of EOCs on structural responses. However, they require synchronized monitoring data covering environmental, operational, and structural conditions. Modeling based on relative features reduces the reliance on complete measurements of environmental and operational parameters, although its applicability requires further validation.

### 4.4. Impact of Sparse Sensing

Spatially sparse sensing limits the observability of structural responses. Insufficient or poorly positioned sensors may fail to capture local damage or certain structural modes because their responses are weak at the measured locations. Responses at uninstrumented locations must therefore be reconstructed using spatial interpolation, modal expansion, or virtual sensing. Interpolation uncertainty increases with sensor spacing and is particularly significant near structural discontinuities and outside the instrumented region. Model-based reconstruction introduces further uncertainty from measurement noise, model errors, and assumptions about modal properties and spatial correlations. Tarpø et al. noted that measurement and modeling errors can propagate into virtual strain estimates [176]. Reconstructed responses should therefore be validated against independent measurements and accompanied by uncertainty bounds where feasible.

Temporal undersampling leads to aliasing when the sampling frequency is less than twice the highest frequency of interest. Aliasing may generate false spectral peaks, bias modal and load estimates, and obscure transient or damage-sensitive responses. The sampling rate should therefore be selected according to the target frequency range, with anti-aliasing filtering applied before downsampling [177]. Spatial sensor placement and temporal sampling should be jointly designed to ensure adequate observability of the monitored responses.

## 5. Artificial Intelligence Methods

### 5.1. Machine Learning Methods

ML is currently one of the most widely adopted data-driven approaches for analyzing OWT sensor data [178]. By learning the correlations among multiple variables, ML algorithms can establish normal behavior models, fault identification models, and health state assessment models, thereby maximizing the utilization of sensor data. Depending on whether the training samples are labeled, these methods are generally categorized into supervised and unsupervised learning [33,34].

Supervised learning establishes input-output mapping relationships using known state labels or historical fault samples, representing the most mature approach applied in wind turbine fault early warning and condition assessment. In early research, Zaher et al. [179] utilized SCADA data to perform automated anomaly identification, demonstrating the engineering feasibility of online fault detection based on historical operational data. Subsequently, Schlechtingen et al. [180] constructed a normal behavior model based on SCADA signals, using prediction errors as the anomaly criterion, which laid the foundation for data-driven CM. Focusing on generator temperature, Guo et al. [181] proposed a trend analysis method based on nonlinear state estimation techniques, which identifies early anomalies through changes in temperature residuals. Li et al. [182] further compared the performance of support vector regression, neural networks, extreme learning machines, and deep belief networks in generator bearing temperature monitoring, revealing that ML models based on temperature time series can effectively support the health condition assessment of key components. To address the challenge of identifying multiple cracks at different positions on wind turbine blades, Ogaili et al. [183] proposed a diagnostic framework based on vibration signals and ML. The combination of ReliefF and K-Nearest Neighbors achieved a classification accuracy of up to 97% under various fault conditions. This shows that traditional supervised learning also has good potential in blade multi-fault identification. Overall, the advantage of supervised learning is that the approach provides a clear model and a relatively mature path for engineering implementation. It is suitable for temperature monitoring, state classification, and fault early warning of components such as gearboxes, generators, and bearings.

Unlike supervised learning, unsupervised learning does not rely on extensive fault labels, making it particularly well-suited for OWT applications characterized by abundant normal samples and scarce fault data. Existing reviews [184] highlight that clustering is one of the most critical approaches for SCADA data anomaly detection. Liu et al. [185] proposed a SCADA anomaly detection framework based on K-means clustering, which initially clusters attributes and subsequently integrates dimensionality reduction and classification strategies to enhance anomaly detection performance. Morrison et al. [186] compared the performance of various anomaly detection methods for power curve cleaning, demonstrating that unsupervised techniques are crucial for identifying abnormal operating points and improving subsequent modeling quality. Campoverde-Vilela et al. [187] constructed a main bearing anomaly detection model based on PCA, successfully achieving early warnings for main bearing faults using exclusively SCADA data. Similarly, Beretta et al. [188] proposed an ensemble learning scheme for the predictive maintenance of main bearings. Although unsupervised learning generally struggles to provide explicit fault classifications, it offers unique advantages in discovering unknown anomalies, identifying performance degradation, and facilitating early warnings.

### 5.2. Deep Learning Methods

Compared with traditional ML methods, deep learning offers superior capabilities in processing highly nonlinear, long time-series, and unstructured data. For OWTs, hydrodynamic behaviors of motions and vibrations under complex environmental conditions exhibit pronounced multi-scale and nonlinear characteristics, which traditional models often struggle to fully capture. By enabling precise feature extraction, deep learning (DL) reduces reliance on manual feature engineering and enhances the representational capacity for high-frequency dynamic responses, images, and multi-source fusion data. In recent years, numerous studies on wind energy systems have demonstrated that DL has expanded its applications from fault diagnosis to complex tasks such as virtual sensing, life prediction, and wind farm predictive maintenance. The most popular types of DL are RNNs and CNNs.

RNNs and their variants, such as Long Short-Term Memory (LSTM) and Gated Recurrent Unit (GRU) networks, excel at processing time-dependent sensor sequences and are consequently extensively applied in predicting the RUL and conducting temporal health assessments for wind turbines. Wang et al. [189] proposed a pre-interaction LSTM method to predict the RUL of wind turbine gearbox bearings under small-sample conditions, demonstrating the robust potential of LSTM-based methods in fatigue life modeling. Similarly, Manna et al. [190] integrated LSTM networks with Monte Carlo dropout techniques to formulate a probabilistic RUL prediction and maintenance planning framework, highlighting the extended utility of deep temporal models in predictive maintenance. For OWTs, particularly FOWTs, long-term temporal modeling extends beyond direct life extension estimation to facilitate the estimation of unmeasurable structural loads and fatigue damage using accessible platform motion and environmental data sequences. Gräfe et al. [191] utilized platform motions and nacelle LiDAR-measured wind field data as neural network inputs to successfully estimate mooring fairlead tensions and fatigue damage equivalent loads. Branlard et al. [192] validated a DT framework on the full-scale TetraSpar floating prototype, demonstrating that operational measurements and sensor data can accurately estimate wind speeds, aerodynamic loads, and tower loads, thereby enabling comprehensive fatigue life analyses.

CNNs and their derivatives exhibit exceptional performance in image and video processing tasks, forming the crucial foundation for automated OWT inspections. Shihavuddin et al. [193] pioneered the application of deep learning in the analysis of UAV blade inspection images, verifying the feasibility of automated structural surface damage identification. Building upon this, Qiu et al. [194] proposed a You Only Look Once (YOLO)-based small object detection approach. They utilized it to identify micro-scale defects, including blade cracks, oil stains, and structural inclusions. This method achieved an average detection accuracy of 91.3%. Fu et al. [195] proposed a lightweight and efficient YOLO model based on an improved YOLO-v7, achieving an effective balance between detection accuracy and real-time inference speed. Overall, CNNs have driven a profound paradigm shift in OWT inspections, transitioning from manual visual checks to automated identification, and from single-modality observations to multimodal data fusion.

Beyond RNNs and CNNs, recent deep-learning studies have increasingly focused on integrating spatiotemporal information, transfer learning, and few-shot learning. Liu et al. [196] used a spatiotemporal graph neural network to model interdependencies within multivariate SCADA data, enabling early anomaly detection in wind turbines. Wang et al. [197] incorporated an LSTM network into a Transformer encoder and combined it with transfer learning to improve the adaptability of a gearbox anomaly early-warning model. To overcome the scarcity of fault samples, Qiao et al. [198] proposed a model-agnostic-meta-learning-based fault diagnosis method for wind turbine generators. Validation results showed that the method maintained good fault-recognition performance and new-task adaptability under limited-sample conditions.

In conclusion, AI techniques have profoundly transformed the processing paradigms for OWT sensor data. Traditional ML algorithms remain highly suitable for structured, low-frequency data exhibiting strong steady-state characteristics, offering high technological maturity in normal behavior modeling, anomaly detection, and early warning systems for critical components. Conversely, deep learning models demonstrate superior representational capabilities in complex domains, such as long-term temporal modeling, virtual sensing, visual inspection, and acoustic monitoring. A structured comparison of representative AI methods for OWT monitoring is provided in Table 4. The qualitative ratings, particularly those for training cost and real-time suitability, are intended as general references because they depend on the specific monitoring task, dataset size, model architecture, and computing platform.

Nevertheless, AI applications still encounter significant bottlenecks, including insufficient model generalization, a severe scarcity of fault labels, the detrimental impacts of sensor drift and missing data, and a fundamental lack of model interpretability. Accordingly, recent advances in AI methods have increasingly focused on improving model robustness, transferability, and trustworthiness in complex marine environments, rather than solely pursuing higher recognition accuracy. Future advancements will likely shift away from purely data-driven approaches that are decoupled from underlying physical mechanics. Instead, the most promising direction lies in the deep integration of physical constraints, domain knowledge, data-driven algorithms, and DT technology to establish a comprehensive hybrid intelligent framework for the full-lifecycle management of offshore wind platforms [199,200].

## 6. Digital Twin Technology

**Figure 5:**
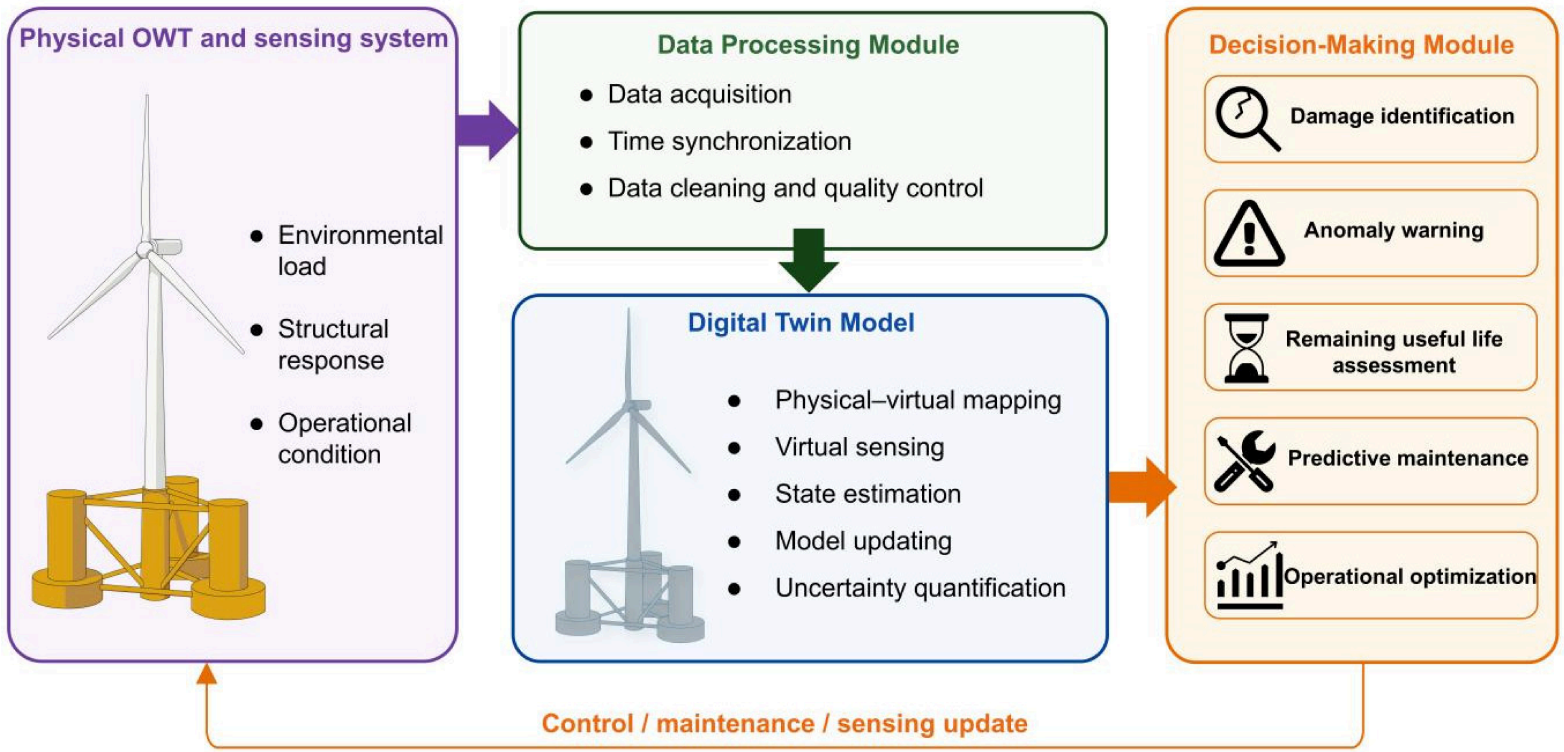
Schematic framework of digital twin (DT)-based structural health monitoring (SHM) for offshore wind turbines (OWTs).

Under the background of the deep integration of modern industry and information technology, the SHM of offshore wind power is experiencing a paradigm shift from traditional data acquisition to intelligent condition perception. In this process, DT technology, as a core technology integrating mechanism models and data-driven methods, provides a brand-new means for the health assessment of wind turbines [201,202]. The essence of DT is the bidirectional mapping and real-time interaction between physical entities and virtual models [203,204]. Figure 5 schematically illustrates the relationships among sensing systems, data processing, virtual-model updating, and decision-making within a DT-based OWT SHM framework. By assimilating real-time data from on-site sensor networks, historical operational logs, and underlying physical mechanisms (e.g., aerodynamics, hydrodynamics, and structural dynamics), DT could construct high-fidelity models capable of dynamically reflecting the actual operational conditions of the turbines [36,205,206]. DT has now become important technical support for the predictive maintenance and full life cycle management of OWTs [207,208,209].

Constrained by installation costs and the harsh marine environment, physical sensors on OWTs are typically deployed in a sparse and discrete manner, resulting in limited and uncertain monitoring information. To overcome this limitation, DT technology leverages high-fidelity finite element and multi-body dynamics models to construct virtual sensors [210,211]. For instance, Ibrahim et al. [212] proposed a DT-based virtual wind speed sensing system by constructing a physical testbed and a corresponding virtual model of a direct-drive permanent magnet synchronous generator. Using a low-latency data acquisition system to synchronize easily measurable electrical parameters in real time, they inversely estimated the effective wind speed required to generate the observed mechanical power. Similarly, relying on a unified linear input and state estimator during a scaled wind turbine test, Zhu et al. [213] successfully predicted the displacement responses of unmonitored nodes and the dynamic loads at the nacelle interface using only partial strain and acceleration data from the tower.

Based on the measured dynamic responses transmitted via low-latency communication links, the DT system leverages algorithms such as Bayesian inference, particle filtering, or ML to perform continuous data synchronization and model updating. By continuously updating key parameters in terms of material degradation properties, boundary constraint stiffness, and damping ratios in real time, the DT model can accurately reflect the current health condition of the structure. For instance, Yu et al. [172] validated the model updating of a 5.5 MW OWT support structure using Bayesian inference, while Rao et al. [68] employed Bayesian spectral decomposition techniques to perform operational modal identification, effectively quantifying parameter uncertainties.

Although DT technology has been recognized to have significant potential in the operation of OWTs with localized validation, it still faces critical technical challenges. Solving the high-fidelity physical models constructed within DT frameworks demands substantial computational resources. This has a serious conflict with the real-time response required by the DT. Consequently, in the actual deployments, model accuracy is frequently sacrificed to ensure adequate computational efficiency. At the same time, developing a customized DT platform requires high costs. Maintaining sensor networks in harsh marine environments is exceptionally costly [214]. This greatly limits the DT technology in wind farms with controllable quality and acceptable costs.

## 7. Conclusions and Future Perspectives

This review has examined the current state-of-the-art in sensing technologies for OWTs. First, it comprehensively outlined the primary monitoring systems utilized in offshore wind farms, encompassing environmental monitoring, SCADA, CMS, and SHM. Second, the uncertainties associated with the sensing technologies were addressed by presenting uncertainty quantification and error correction methods. Finally, it highlighted the prospective applications of advanced AI and DT technologies within the monitoring domain. The primary conclusions drawn from this review are summarized as follows:

Accelerometers and strain gauges are widely used in SHM because of their cost-effectiveness, while FBG sensors are promising for blade strain measurement. Future developments should improve the noise robustness of AE monitoring and the environmental adaptability of visual inspection. Overall, OWT health monitoring is progressing towards full-field, remote, wireless, and real-time monitoring.

Single-sensor measurements cannot fully characterize structural health. Future research should therefore focus on multi-source data fusion. Although DT provides a framework for multimodal data integration, the synchronization and cleaning of large-scale heterogeneous data remain computationally and economically demanding.

Sensor layout should be optimized to balance monitoring effectiveness and the number of sensors. Full-field response reconstruction is also expected to support the future deployment of DT systems.

Sensor degradation caused by salt spray and measurement errors induced by the motion of remote and non-contact sensing platforms require further investigation. Future studies should integrate sensor condition assessment with platform motion compensation and quantify the propagation of measurement uncertainty into fatigue damage assessment and RUL estimation. Unsupervised learning may also help distinguish early damage features from structural responses affected by EOCs.

The engineering application of DL remains constrained by limited labeled damage data. Representative OWT benchmark datasets and standardized validation protocols are therefore needed to support consistent comparison and validation of AI methods. Physics-informed neural networks and transfer learning may further reduce reliance on measured damage samples and improve model applicability to OWTs.

## Figures and Tables

**Table 1:** Summary of commonly used sensors and their characteristics for offshore wind turbines (OWTs).

Category	Monitoring Parameter	Popular Technology	Advantages	Limitations	Target Structure	Ref.
Environmental Monitoring	Wind	Nacelle anemometers	Captures aerodynamic loads and wind shear	Wake interference	Ambient wind field, nacelle	[19,20,21]
		Light Detection and Ranging (LiDAR) systems		Complex motion compensation		[22,23]
	Wave	Wave buoys	Evaluates hydrodynamic loads and wave run-up impacts	Physical dislocation risk	Sea surface, platform air-gap	[24,25,26,27]
		Wave radars		Weather sensitivity		[28,29]
	Current	Acoustic Doppler Current Profilers (ADCPs)	Full-depth velocity profiling for fatigue analysis	Biofouling Acoustic noise Challenging operations and maintenance (O&M)	Subsea flow field	[30,31]
Operating State	Operating Status	Supervisory Control and Data Acquisition (SCADA) systems	Continuous comprehensive operational monitoring Exceptionally low implementation cost	Massive data volumes High false alarm susceptibility Low sampling rates Restricted transient dynamic analysis capability	Entire system, rotor, nacelle	[32,33,34]
Condition Monitoring	Drivetrain Faults	High-frequency accelerometers, oil debris monitors	Proven standard Averts catastrophic failures	High false-positive rates Complex data extraction	Gearbox, generator, bearings	[35,36]
Structural Health Monitoring	Vibration	Micro-Electro-Mechanical Systems (MEMS) accelerometers	Low cost and lightweight Simple operation High technological maturity	Susceptible to external environmental interference Less sensitive to local vibration	Tower, blades, nacelle, foundation	[37,38]
	Strain	Electrical resistance strain gauges	Low cost and lightweight Direct and intuitive measurement	Provides highly localized measurements Poor resistance to electromagnetic interference Prone to zero-drift and aging	Tower base, blade roots, critical joints	[39,40,41]
		Fiber Bragg Grating (FBG) sensors	High sensitivity Excellent electromagnetic interference immunity	High cost Sensitive to temperature fluctuations Stringent installation requirements	Blades, tower	[42,43]
	Crack	Acoustic emission (AE) probes	Broad spatial coverage High measurement accuracy	High cost Inability to detect dormant or non-propagating cracks	Blades, welded joints	[44,45]
	Displacement, Surface Defects	Optical cameras, unmanned aerial vehicle (UAV) platforms	Non-contact inspection High spatial maneuverability Machine vision enabled defect recognition	High dependency on illumination and weather Limited battery endurance Computationally intensive processing	Blades, tower surface	[46]
	Displacement	Global Navigation Satellite System (GNSS) receivers, inclinometers	Direct measurement of absolute positioning and tilt angle	Signal multipath effects Low sampling rate Temperature drift	Tower top, nacelle	[47,48]
	Bolt Loosening	Strain sensors, ultrasonic probes, infrared thermographic cameras, optical sensors	Real-time estimation of residual torque and preload Quantitative assessment of internal and surface defects	Prohibitive economic costs for comprehensive continuous coverage	Tower flanges, blade root connections	[49,50]
	Mooring Tension	Load cells, fiber optic sensors	Real-time tension tracking Reliable station-keeping assessment	Blind spots at intermediate nodes and anchor points Inability to capture sudden tension surges	Mooring lines, anchors, fairleads	[51]

**Table 3:** Quantification and mitigation of sensing-data uncertainty in offshore wind monitoring.

Uncertainty Source	Main Mitigation or Quantification Method	Typical Parameters and Metrics	Advantages	Limitations	Ref.
Measurement noise	Frequency-domain filtering	Cutoff frequency; filter order; SNR; RMS noise	Simple and intuitive; suitable for noise within known frequency bands	Depends on frequency-band selection; may remove valid response components	[152,153]
	Wavelet-threshold denoising	Mother wavelet; decomposition level; ΔSNR; RMSE	Suitable for non-stationary signals; preserves transient features	Parameter selection can be subjective; may cause signal distortion	[152,153]
	CEEMDAN- or VMD-based denoising	Ensemble size/number of modes; noise amplitude/penalty factor; SNR; RMSE	Suitable for non-stationary, multicomponent signals; facilitates frequency-component separation	Computationally intensive; sensitive to parameter selection	[154,155]
Missing measurement data	Statistical- or correlation-based missing-data imputation	Window length; number of neighboring samples; MAE; RMSE	Low computational cost; suitable for short data gaps	Unsuitable for long consecutive gaps; depends on data correlations	[120,138]
Sensor bias and drift	Multivariate statistical analysis, such as PCA, PLS, and residual analysis	Number of principal components; residual threshold; drift rate; reconstruction error	Suitable for multivariate data	Depends on training data; limited generalizability	[153,156,157]
Temperature-induced measurement error	Temperature-coefficient correction; reference-temperature compensation	Temperature coefficient; reference temperature; compensation residual; RMSE	Practical for engineering applications	Requires synchronized temperature data; affected by installation conditions	[158]

Note: CEEMDAN, complete ensemble empirical mode decomposition with adaptive noise; VMD, variational mode decomposition; PCA, principal component analysis; PLS, partial least squares; SNR, signal-to-noise ratio; ΔSNR, improvement in signal-to-noise ratio; RMS, root mean square; MAE, mean absolute error; RMSE, root mean square error.

**Table 4:** Representative AI methods for OWT monitoring (Based on the data from references used in this paper).

Category	Typical Methods	Used Data	Main Applications	Key Features	Data Requirements	Interpretability	Training Cost	Real-Time Suitability	Main Limitations
Supervised Learning	Artificial neural network (ANN)	SCADA; vibration	Fault detection; condition classification	High predictive accuracy	High; labeled health or fault data	Low–Medium	Low–Medium	High after training	Large labeled datasets; sensitivity to class imbalance and domain shifts
Unsupervised Learning	K-means; PCA	SCADA	Anomaly detection; early warning	No labeled fault data required	Medium; representative normal-operation data	Medium	Low	High after model establishment	Threshold sensitivity; false alarms and uncertain physical interpretation
RNN-based Deep Learning	LSTM; GRU	SCADA; vibration; strain	RUL prediction; temporal condition assessment	Strong temporal modeling capability	Medium–High; continuous sequential data	Low	Medium–High	Medium	Sensitivity to missing or irregular sequences; limited transfer across turbines and operating conditions
CNN-based Deep Learning	CNN; YOLO	Images	Structural damage detection	Strong spatial feature extraction	High; labeled image data	Low–Medium	High	Medium–High; model- and hardware-dependent	Dependence on labeled images; sensitivity to illumination, viewpoint, weather, and image quality

## Data Availability

No new data were created or analyzed in this study. Data sharing is not applicable to this article.

## Abbreviations

The following abbreviations are used in this manuscript:

ADCP	Acoustic Doppler Current Profiler
AE	Acoustic Emission
AI	Artificial Intelligence
ANN	Artificial Neural Network
BiLSTM	Bidirectional Long Short-Term Memory
CEEMDAN	Complete Ensemble Empirical Mode Decomposition with Adaptive Noise
CM	Condition Monitoring
CMS	Condition Monitoring System
CNN	Convolutional Neural Network
DL	Deep Learning
DT	Digital Twin
EOC	Environmental and Operational Conditions
EPON	Ethernet Passive Optical Network
FBG	Fiber Bragg Grating
FOWT	Floating Offshore Wind Turbine
GA	Genetic Algorithm
GNSS	Global Navigation Satellite System
GP	Gaussian Process
GRU	Gated Recurrent Unit
IMU	Inertial Measurement Unit
LiDAR	Light Detection and Ranging
LoRa	Long Range
LoRaWAN	Long Range Wide Area Network
LSTM	Long Short-Term Memory
LTE	Long-Term Evolution
MAE	Mean Absolute Error
MEMS	Micro-Electro-Mechanical Systems
ML	Machine Learning
NB-IoT	Narrowband Internet of Things
O&M	Operations and Maintenance
OWT	Offshore Wind Turbine
PCA	Principal Component Analysis
PLS	Partial Least Squares
RMS	Root Mean Square
RMSE	Root Mean Square Error
RNN	Recurrent Neural Network
RUL	Remaining Useful Life
SCADA	Supervisory Control and Data Acquisition
SHM	Structural Health Monitoring
SNR	Signal-to-Noise Ratio
ΔSNR	Improvement in Signal-to-Noise Ratio
TRL	Technology Readiness Level
UAV	Unmanned Aerial Vehicle
VMD	Variational Mode Decomposition
YOLO	You Only Look Once
4G	Fourth Generation
5G	Fifth Generation
